# Predicting the standing stock of organic carbon in surface sediments of the North–West European continental shelf

**DOI:** 10.1007/s10533-017-0310-4

**Published:** 2017-02-15

**Authors:** Markus Diesing, Silke Kröger, Ruth Parker, Chris Jenkins, Claire Mason, Keith Weston

**Affiliations:** 1 Centre for Environment, Fisheries and Aquaculture Science, Lowestoft, Suffolk, NR33 0HT UK; 2 Institute of Arctic and Alpine Research, University of Colorado, Boulder, CO 80309-0450 USA

**Keywords:** Organic carbon, Continental shelf, Sediment, Spatial prediction, Europe

## Abstract

Shelf seas and their associated benthic habitats represent key systems in the global carbon cycle. However, the quantification of the related stocks and flows of carbon are often poorly constrained. To address benthic carbon storage in the North–West European continental shelf, we have spatially predicted the mass of particulate organic carbon (POC) stored in the top 10 cm of shelf sediments in parts of the North Sea, English Channel and Celtic Sea using a Random Forest model, POC measurements on surface sediments from those seas and relevant predictor variables. The presented model explains 78% of the variance in the data and we estimate that approximately 250 Mt of POC are stored in surficial sediments of the study area (633,000 km^2^). Upscaling to the North–West European continental shelf area (1,111,812 km^2^) yielded a range of 230–882 Mt of POC with the most likely estimate being on the order of 476 Mt. We demonstrate that the largest POC stocks are associated with coarse-grained sediments due to their wide-spread occurrence and high dry bulk densities. Our results also highlight the importance of coastal sediments for carbon storage and sequestration. Important predictors for POC include mud content in surficial sediments, annual average bottom temperature and distance to shoreline, with the latter possibly a proxy for terrestrial inputs. Now that key variables in determining the spatial distribution of POC have been identified, it is possible to predict future changes to the POC stock, with the presented maps providing an accurate baseline against which to assess predicted changes.

**Electronic supplementary material:**

The online version of this article (doi:10.1007/s10533-017-0310-4) contains supplementary material, which is available to authorized users.

## Introduction

Carbon dioxide (CO_2_) from the atmosphere is taken up by seawater, where it is fixed by primary producers such as phytoplankton with a proportion of this particulate organic carbon (POC) supporting the food web within the water column, while another part sinks to the seabed (Miller 2004). The latter may be incorporated into the surface sediments or support the benthic food web and be respired or buried, along with POC from terrestrial sources. The sedimentation of POC is therefore a key process in transferring CO_2_ from the atmosphere to the seabed where it may be stored long term (decades–centuries) mitigating increases in atmospheric CO_2_ associated with climate change (Pachauri and Meyer 2014). There are, however, many natural mechanisms that affect the incorporation of POC into sediments, from physical processes such as particle movement or bedform migration due to storms and tides (Jenness and Duineveld 1985) or water column temperature (Berner 1980; Middelburg 1989 and references there-in), to biological processes such as infaunal activity and mode of feeding (Aller 1982). POC supply, incorporation and storage may also be perturbed by human activities such as bottom trawling (Duplisea et al. 2001; Trimmer et al. 2005) through direct mixing or indirect impact on the infaunal community. These processes can also be altered through changes in supply through localised eutrophication or redox effects associated with differing bottom oxygen regimes and anoxia (Diaz and Rosenberg 2008; Middelburg and Levin 2009). Ultimately, it is the balance between the supply from the water column and remineralisation rate of benthic POC, which controls the POC stock in the surficial sediments and which will dictate the incorporation rate of POC. An increasing POC stock, either over space or time, therefore implies an increasing input or decreasing remineralisation. This may be due to either changing/contrasting lability of POC (i.e. terrestrial vs marine source), natural conditions or human pressures (Burdige 2005, 2007).

To understand how this range of processes affects POC storage and hence how they will be affected under future change or human pressure conditions, it is first necessary to quantify the stock. Generally, these natural and anthropogenic processes affect the upper sediment layer and this study therefore estimates the amount of POC in the surface sediments (0–10 cm) of the North-West (NW) European continental shelf using a data set of directly sampled stations to better constrain their POC stocks and hence their role in the carbon cycle. Understanding the functioning and distribution of these coastal and shelf sea POC stores is also critical since there are considerable pressures on these systems both from local and far field effects (Bauer et al. 2013) and by improving our quantification and the underpinning conditions associated with changes in the levels of such stores, we can better understand the effects of future changes to them and in turn the global carbon cycle.

Data on POC measurements have been reported in the literature. These datasets are largely a result of detailed, small scale studies on sediment biogeochemical processing of carbon and associated pathways or linked to faunal community analysis (Basford et al. 1993; Willems et al. 2007; Stockdale et al. 2009 and references there-in). As a result, comparatively few attempts have been made so far to spatially predict POC in surficial sediments across defined areas of seabed: Mollenhauer et al. (2004) presented a map of organic carbon in surface sediments of the South Atlantic Ocean based on 1118 samples; Seiter et al. (2004) spatially predicted organic carbon content in the top 5 cm of global deep sea sediments at a 1º × 1º grid resolution by the application of a combined qualitative and quantitative-geostatistical method; Acharya and Panigrahi (2016) mapped the distribution of organic carbon on the Eastern Arabian shelf with Empirical Bayesian Kriging; Serpetti et al. (2012) mapped the organic content of coastal sediments from hydro-acoustic reflectance data in an area of seabed off the east coast of Scotland; and Neto et al. (2016) assessed the suitability of seismic peak amplitude as a predictor of total organic carbon content in shallow marine sediments, based on data collected in the Cabo Frio mud belt in an upwelling zone off south-eastern Brazil. This indicates that maps of POC concentration in surficial sediments have either been derived by means of acoustic methods (Serpetti et al. 2012; Neto et al. 2016) with limited spatial coverage or some type of kriging (Mollenhauer et al. 2004; Seiter et al. 2004; Acharya and Panigrahi 2016).

More recently, machine learning algorithms have made inroads into spatial prediction and have been used to spatially predict categorical (e.g. sediment types; Stephens and Diesing (2014)) and continuous (e.g. sediment composition; Stephens and Diesing (2015)) data. Machine learning algorithms are data-driven flexible statistical prediction techniques that ‘learn’ patterns in data to predict an associated value. Machine learning is defined as “programming computers to optimise a performance criterion using example data or past experience” (Alpaydin 2010). Such predictive mapping methods entail a two-step approach: Initially, the relationship between a set of predictor variables and a response variable is modelled from observations (samples). The established model is then employed to predict the response variable at unsampled locations for which values of the predictor variables are known.

The aim of this study is to map and quantify POC in surficial sediments over a large area. Hence, acoustic methods are not applicable due to the lack of suitable data. Geostatistical (kriging) methods would be applicable in this case. However, there are advantages in using a machine learning approach for the task in hand, as such methods do not need to satisfy strict statistical assumptions as is the case for kriging. Additionally, such methods allow investigation of predictor-response variable relationships, which might shed light on factors and processes controlling POC in surficial sediments at a regional scale, in contrast to site specific studies undertaken previously.

The objectives of this paper are to (i) develop a machine learning methodology that allows to spatially predict POC concentrations in surficial sediments of the NW European continental shelf in an accurate and validated way, (ii) estimate the mass of POC stored in surface sediments and (iii) elucidate relationships between POC concentrations and relevant environmental variables.

## Regional setting

**Fig. 1:**
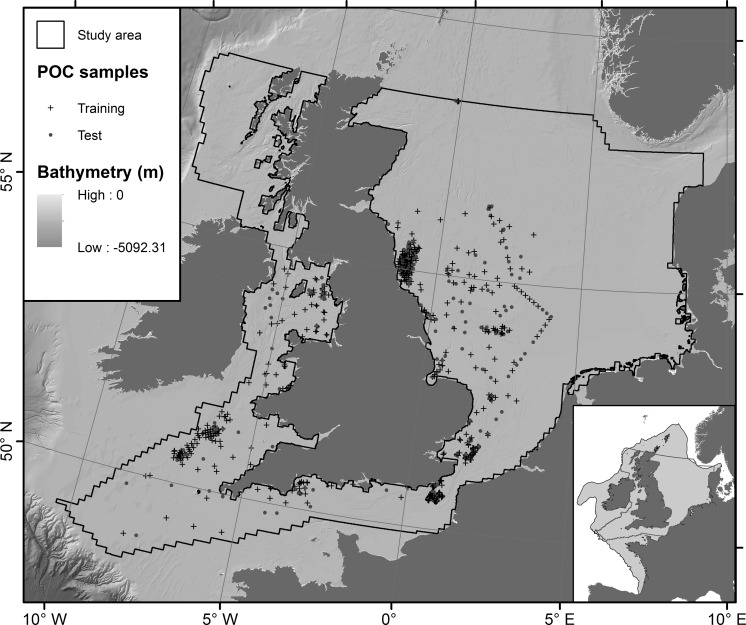
Location of the study area on the NW European continental shelf (*inset*). Also shown are the locations of POC samples, split into training and test datasets

The study area is part of the NW European continental shelf adjacent to the North–East Atlantic Ocean. It includes parts of the North Sea, English Channel and Celtic Seas (Fig. 1) and measures approximately 633,000 km^2^. Water depths range between 0 and 1235 m below sea level, with a mean depth of 67 m. Most of the area (98.6%) is continental shelf with depths shallower than 200 m. Within the study area, greater water depths are only found in the Norwegian Trench and on the continental slope off the Scottish West coast.

## Data

### Response variable

A total of 1111 measurements of the concentration of POC in the sediment fraction <2 mm collected between 1996 and 2015 were collated from the Centre for Environment, Fisheries and Aquaculture Science (Cefas) in-house data holdings (Mason et al. 2017). Sediment samples were freeze-dried and any material >2 mm was removed. The sediment was subsequently ground and inorganic carbon removed using a sulphurous acid digest. POC concentrations were measured using an elemental analyser. For some sites, more than one measurement of POC concentration existed in the database. These were either repeat measurements (replicates) or measurements for different depth horizons (0–5 cm and 5–10 cm). Average values of POC concentration were calculated in such instances, which reduced the number of records to 1004. Of those, 849 records co-occurred with all available predictor variables (see below) and these were used for further analysis. The statistics of POC concentrations for varying depth intervals are shown in Table 1.

**Table 1:** Statistics of POC concentration by depth interval

Depth	N	Mean (%)	SD (%)	Min (%)	Max (%)
Surface (nominally 0–2 cm)	711	0.46	0.51	0.02	4.49
Surface layer; variable depths; max depth = 5 cm	33	0.21	0.17	0.03	0.70
0–5 cm	33	0.22	0.20	0.03	1.00
0–10 cm	72	0.52	0.36	0.07	1.64

The POC concentrations are reported as a proportion (weight-%). In such a case, an arcsine transformation is advisable (Sokal and Rohlf 1981):

1 $$Y = \arcsin \surd X,$$

with X being measured POC as a fraction (ranging from 0 to 1) and Y being the transformed POC concentration. Back-transformation of the predicted values is achieved via:

2 $$X = (\sin Y)^{2}$$

The data set was randomly split into training and test data with a ratio of 2:1 respectively, yielding 566 samples for model training and 283 samples for model testing.

### Predictor variables

Predictor environmental variables were initially selected based on their expected relevance to the spatial distribution in POC and their availability. The predictor variables are comprised of bathymetry, Euclidean distance to the nearest shoreline, geographic position (eastings, northings), sediment composition (mud, sand and gravel fraction), earth observation data (chlorophyll-a, depth of the euphotic zone and suspended particulate matter (SPM) concentrations) from the moderate resolution imaging spectroradiometer (MODIS), hydrodynamic model data (depth averaged mean and peak current speed, peak wave orbital velocity and peak wave-current shear stress), water-column bottom salinity (annual average and range), water-column bottom temperature (annual average and range) and stratification (thermal and salinity).

## Methods

### Random forest regression

The random forest (RF) prediction algorithm (Breiman 2001) was chosen as the modelling tool for the analysis because it has shown high predictive accuracy in a number of domains (Prasad et al. 2006; Huang et al. 2012; Mutanga et al. 2012; Oliveira et al. 2012; Huang et al. 2014). RF can be used without extensive parameter tuning, it can handle a large number of predictor variables, is insensitive to the inclusion of some noisy/irrelevant features, makes no assumptions regarding the shape of distributions of the response or predictor variables (Cutler et al. 2007) and is therefore suitable for this analysis. The RF is an ensemble technique that ‘grows’ many regression trees. It is called a random forest because two elements of randomness are introduced. Firstly, each tree is constructed from a bootstrapped sample of the training data. Secondly, only a random subset of the predictor variables is used at each split in the tree building process. This has the effect of making every tree in the forest unique. The underlying principle of the technique is that although each tree in the forest may individually be a poor predictor and that any two trees could give different answers, by aggregating the predictions over a large number of uncorrelated trees, prediction variance is reduced and accuracy improved (James et al. 2013: p. 316). Observations not included in each tree construction, the ‘out-of-bag’ (OOB) samples, are then used to create a form of cross-validated prediction error. RF also provides a relative estimate of predictor variable importance. This is measured as the relative increase in mean squared error associated with each variable when it is assigned random but realistic values and the rest of the variables are left unchanged. The randomForest package (Liaw and Wiener 2002), executed via the Marine Geospatial Ecology Tools v08a.58 (Roberts et al. 2010), was used for the implementation of the model. The forest had 500 trees, and the number of variables tested at each split equalled the number of predictor variables divided by three (rounded down). These are the default settings, which were selected as an increase in the number of trees or variables tested at each split did not lead to improved results (measured as variance explained, see below).

### Model validation

The RF implicitly carries out a form of cross-validation (CV) using the OOB observations. This usually gives a reliable measure for real model performance assuming enough trees are grown (Liaw and Wiener 2002). In addition to this performance indicator, the model constructed here is tested against the test set of observations. For both the CV and the test set, the performance is assessed by calculating the mean of the squared prediction error:

3 $$MSE_{{\hat{y}}} = \frac{1}{n}\mathop \sum \limits_{i = 1}^{n} \left( {y_{i} - \hat{y}_{i} } \right)^{2}$$

where *y* are observed and $\hat{y}$ are predicted values. The ‘variance explained’ (VE) by the model is then calculated by taking the ratio of the MSE to the variance (*σ* ^2^) of the observed values:

4 $$VE = 1 - \frac{{MSE_{{\hat{y}}} }}{{\sigma_{y}^{2} }}$$

The predictions of the transformed and back-transformed response variable were compared with the observed values from the test set and Pearson product-moment correlation coefficients calculated.

### Variable selection

Variable selection reduces the number of predictor variables to a subset that is relevant to the problem. The aims are to reduce redundancy without losing information content and to increase the interpretability of the model. Predictor variables were selected in a two-step approach: Initially, the Boruta variable selection wrapper algorithm (Kursa and Rudnicki 2010) was employed to identify important predictor variables. Wrapper algorithms identify relevant features by performing multiple runs of predictive models, testing the performance of different subsets (Guyon and Elisseeff 2003). The Boruta algorithm creates copies of all variables and randomises them. These so-called shadow variables are added to the predictor variable data set and the RF algorithm is run to compute variable importance scores for predictor and shadow variables. The maximum importance score among the shadow variables (MZSA) is determined. For every predictor variable, a two-sided test of equality is performed with the MZSA. Predictor variables that have a variable importance score significantly higher than the MZSA are deemed important. Likewise, predictor variables that have a variable importance score significantly lower than the MZSA are deemed unimportant. Tentative variables have a variable importance score that is not significantly different from the MZSA. Second, a RF model was run with the remaining predictor variables to establish the variable importance. Beginning with the most important variable, correlated variables (|r| > 0.5) with lower importance were subsequently removed.

### Partial dependence plots

Partial dependence plots (Hastie et al. 2009: pp. 369–370) give a graphical depiction of the marginal effect of a predictor variable on the response. They allow to visualise the effect of a predictor variable on the response variable, while averaging out the effects of all other predictors. Partial dependence plots are a useful tool for data exploration. We used the Marine Geospatial Ecology Tools v08a.58 (Roberts et al. 2010) to create partial dependence plots for selected predictor variables.

### Estimation of dry bulk density

Porosity (ϕ) of the surficial sediment layer was derived from predicted mud content (Stephens and Diesing 2015) employing an equation from Jenkins (2005):

5 $$\phi = 0.3805 \cdot C_{mud} + 0.42071,$$

with ϕ and *C*_*mud*_ (mud content) both given as dimensionless fractions. The equation is based on data from the Mississippi–Alabama–Florida shelf. By applying this equation to our study area, we assumed that the relationship is not site specific. To test the validity of this assumption, we compared estimates made this way with porosity measurements carried out at 55 stations in the Celtic Sea (Silburn et al. 2017). We also apply an alternative mud-porosity relationship based on the same porosity measurements and predicted mud content (Stephens and Diesing 2015) to assess the differences in the estimation of porosity.

Dry bulk density (ρ_d_) of the sediment was then derived from sediment porosity and grain density (ρ_s_ = 2650 kg m^−3^) according to:

6 $$\rho_{d} = \left( {1 - \phi} \right)\rho_{s}$$

All calculations were carried out in ArcGIS v10.1 using the Raster Calculator tool.

### Estimation of the total mass of POC stored in surficial sediments

The mass of POC (m_POC_) per grid cell was calculated by multiplying POC concentration (as a dimensionless fraction) with dry bulk density (in kg m^−3^), the sediment depth (d = 0.1 m) and the area of the grid cell (A = 250,000 m^2^):

7 $$m_{POC} = POC \cdot \rho_{d} \cdot d \cdot A$$

A summation of the values of all grid cells yielded the total standing stock of POC.

### Calculation of statistics for POC and dry bulk density per sediment class

Statistics of POC concentrations and dry bulk density were calculated for Folk sediment classes as mapped by Stephens and Diesing (2015). For each sediment class, 1000 random samples were generated and the predicted values of POC concentration and dry bulk density extracted. Due to low spatial extent, the number of samples was lower for some classes (gravelly mud, muddy gravel and slightly gravelly sandy mud). The mean, standard deviation, 5th and 95th percentile of POC concentrations and dry bulk density were calculated.

### Scaling up to the NW European continental shelf

Results from the study area were scaled up to the NW European continental shelf (Fig. 1, inset) in two ways: First, we calculated m_POC_ by multiplying the area of the NW European continental shelf (A = 1,111,812 km^2^) with the average mass of POC per m^2^ and to a depth of 0.1 m as derived for the study area. Second, we employed statistics on POC and dry bulk density and estimates of spatial extent for the different Folk sediment classes (Supplement 1). We used mean values of POC and dry bulk density to derive an estimate of m_POC_. To account for uncertainty in scaling up our results, we calculated estimates from the extreme low and high end of the distributions of POC and dry bulk density. We used the 5th and 95th percentiles to avoid influence of outliers in either direction.

## Results

### Variable selection and importance

**Fig. 2:**
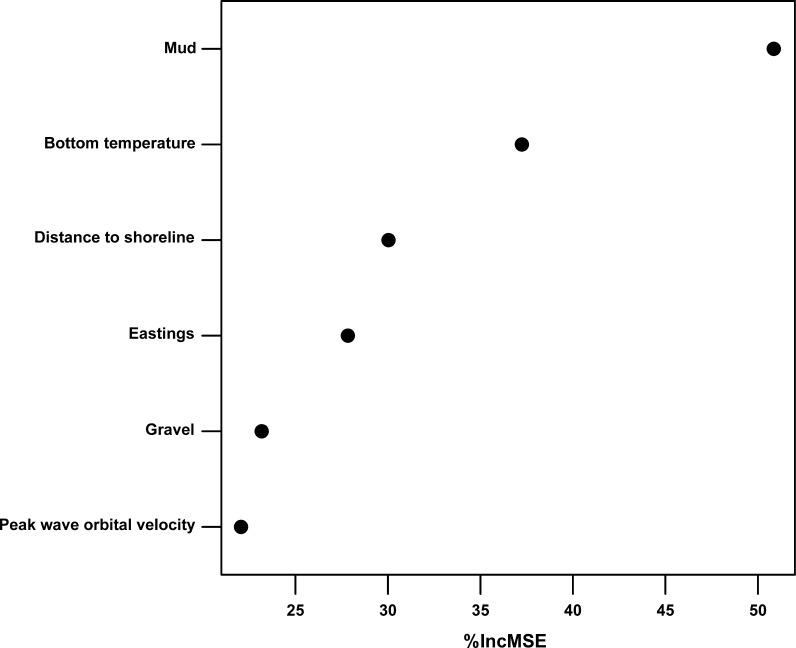
Variable importance scores. The importance of predictor variables as indicated by the random forest algorithm. The *x*-*axis* indicates the relative increase in mean squared error when the variable is assigned random but realistic values, the *y*-*axis* indicates the variables of the final model

The Boruta variable selection process indicated that nine variables were deemed important (Table 2). Subsequent removal of correlated variables reduced the number of variables to six. The final selected variables were mud content, annual average water column bottom temperature, eastings, distance to shoreline, gravel content and peak wave orbital velocity. Figure 2 shows the relative importance of the six variables to prediction accuracy. Mud content in surface sediments is the most important variable in predicting POC, followed by the annual average water column bottom temperature (Table 3).

**Table 2:** List of predictor variables, results of the Boruta variable selection process and final selection of variables after removal of correlated variables

Predictor variable	Boruta	Final	Source
Bathymetry	Tentative		EMODnet-Bathymetry (http://www.emodnet-bathymetry.eu/), Astrium Oceanwise (2011)
Distance to shoreline	Important	Selected	Calculated
Eastings	Important	Selected	Calculated
Northings	Tentative		Calculated
Mud	Important	Selected	Stephens and Diesing (2015)
Sand	Important		Stephens and Diesing (2015)
Gravel	Important	Selected	Stephens and Diesing (2015)
Chlorophyll-a	Tentative		Gohin et al. (2005)
Depth of euphotic zone	Unimportant		Gohin et al. (2005)
SPM (Winter)	Unimportant		Gohin et al. (2005)
SPM (Summer)	Unimportant		Gohin et al. (2005)
Average current speed	Tentative		Aldridge et al. (2015), Bricheno et al. (2015)
Peak current speed	Important		Aldridge et al. (2015), Bricheno et al. (2015)
Peak wave orbital velocity	Important	Selected	Aldridge et al. (2015), Bricheno et al. (2015)
Peak wave-current stress	Tentative		Aldridge et al. (2015), Bricheno et al. (2015)
Annual average bottom salinity	Tentative		Berx and Hughes (2009)
Annual amplitude bottom salinity	Important		Berx and Hughes (2009)
Annual average bottom temperature	Important	Selected	Berx and Hughes (2009)
Annual amplitude bottom temperature	Tentative		Berx and Hughes (2009)
Stratification salinity	Unimportant		Calculated from Berx and Hughes (2009)
Stratification temperature	Tentative		Calculated from Berx and Hughes (2009)

**Table 3:** Cross-validation and test set performance

Statistic	Value
MSE (cross-validation)	0.000273
MSE (test set)	0.000273
Variance explained (cross-validation)	74.9%
Variance explained (test set)	77.5%

### Model validation

**Fig. 3:**
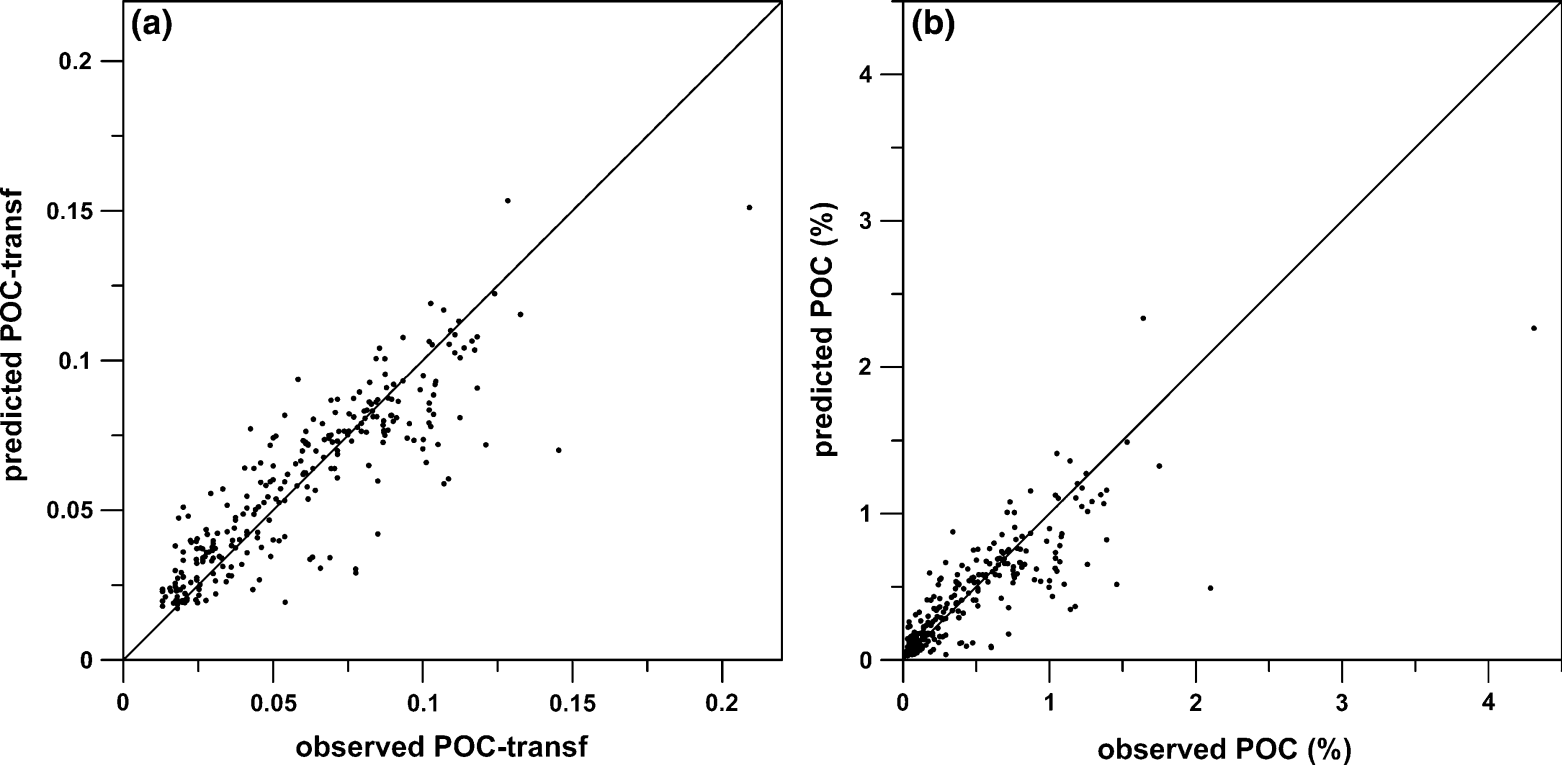
Observed versus predicted values for transformed POC (**a**) and POC concentrations (**b**). The *diagonal line* indicates y = x

Approximately 75% of the variability of the transformed POC values is explained by the RF model (Table 3). The good agreement between cross-validated and test set statistics indicates that the model is not over-fitted to the training data and it is generalising real patterns in the data. Figure 3 shows the predicted versus the observed values for the transformed POC (left) and POC concentration (right). From this it is apparent that the model tends to slightly under-predict POC concentration (predicted POC = 0.7896 * observed POC when forced through origin). Pearson product-moment correlation coefficients for the transformed POC and POC concentration are r = 0.880 (n = 275, p < 2.2e−16) and 0.842 (n = 275, p < 2.2e−16), respectively.

### Data exploration

**Fig. 4:**
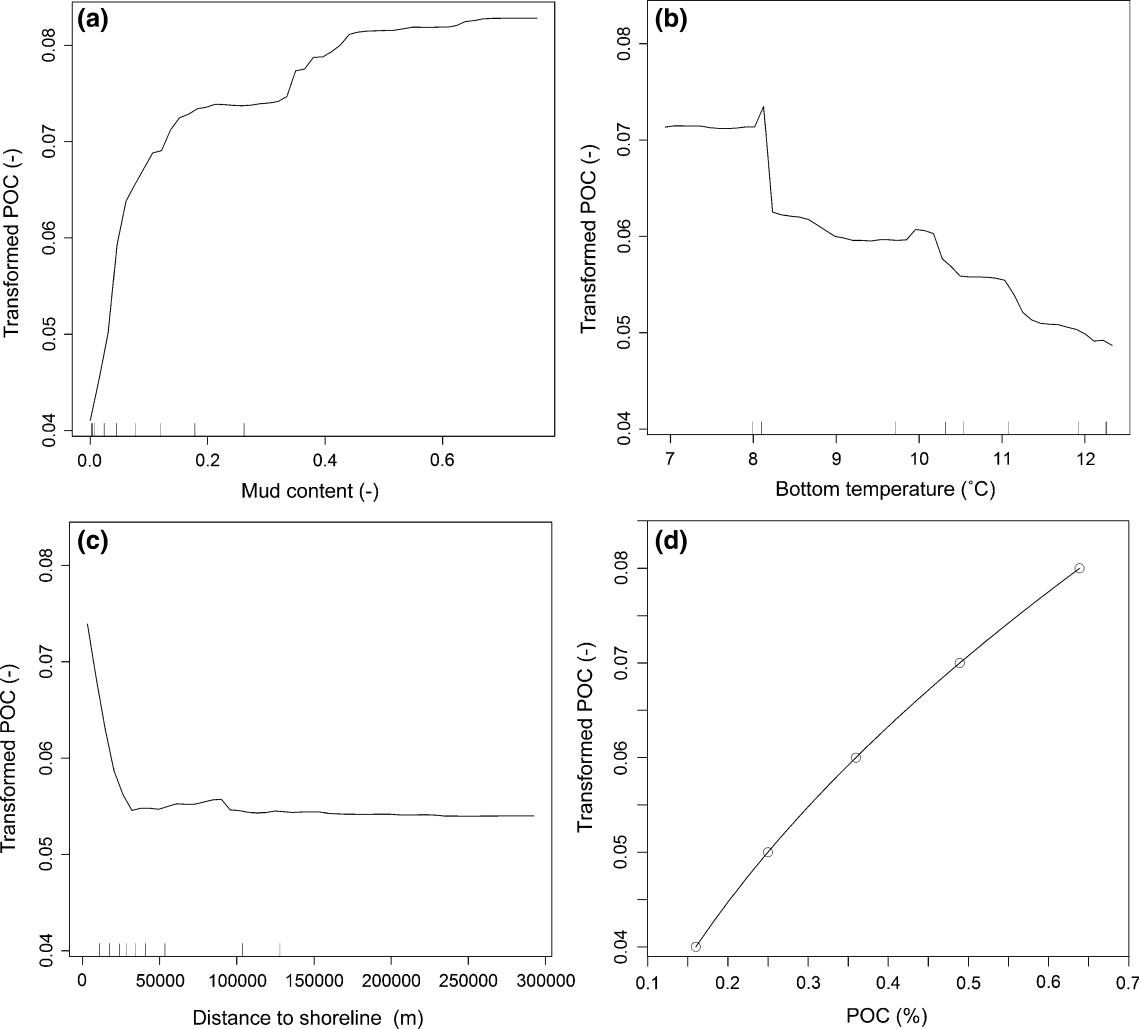
*Partial dependence plots* showing the relationships between mud content (**a**), annual average water column bottom temperature (**b**), distance to shoreline (**c**) and transformed POC. Also shown is the relationship between transformed POC and POC (**d**)

Partial dependence plots (Fig. 4) allow graphical exploration of the relationships between the response variable (transformed POC) and selected predictor variables. Also shown is the relationship between transformed POC and POC (Eq. 1) to aid the interpretation. Transformed POC, and likewise POC, increases with an increasing mud content; however, this increase is not uniform and levels off towards higher mud content of >0.5 (i.e. 50 weight-%). For bottom water-column temperatures below approximately 8 °C transformed POC stays constant at around 0.072 (0.52% POC), then drops steeply to 0.063 (0.4% POC). Beyond that, increasing bottom temperatures relate to a broadly linear decrease in transformed POC. Transformed POC values drop sharply with increasing distance from the shoreline up to about 30 km, then stay broadly constant at about 0.055 (0.3% POC).

### Estimates of porosity and dry bulk density

**Fig. 5:**
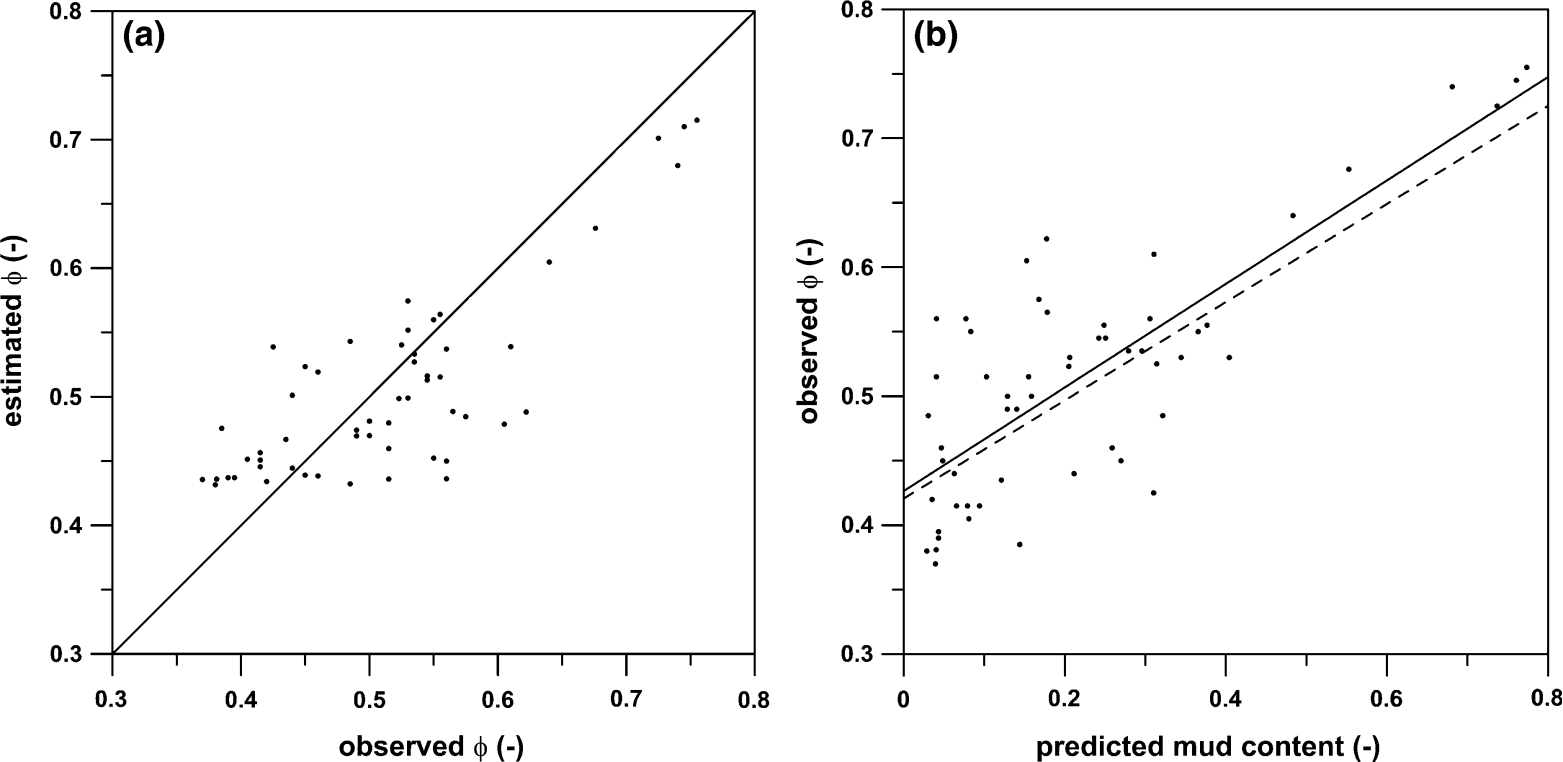
**a** Observed versus estimated values of sediment porosity (ϕ). The *diagonal line* indicates y = x. **b** Predicted mud content versus observed porosity. The *solid line* indicates the best fit linear regression (Eq. 8), the *dashed line* indicates Eq. 5

Figure 5a shows values of measured porosity plotted against estimates of porosity derived from predicted mud content (Stephens and Diesing 2015) using Eq. 5. Both are strongly (Pearson product-moment correlation coefficient, r = 0.803) and significantly (p = 1.65e−13) correlated and points plot along the diagonal line that indicates perfect agreement. Plotting measured porosity against predicted mud content (Fig. 5b) yields a relationship that closely resembles Eq. 5:

8 $$\phi = 0.4013 \cdot {\text{mud }} + 0.4265$$

**Fig. 6:**
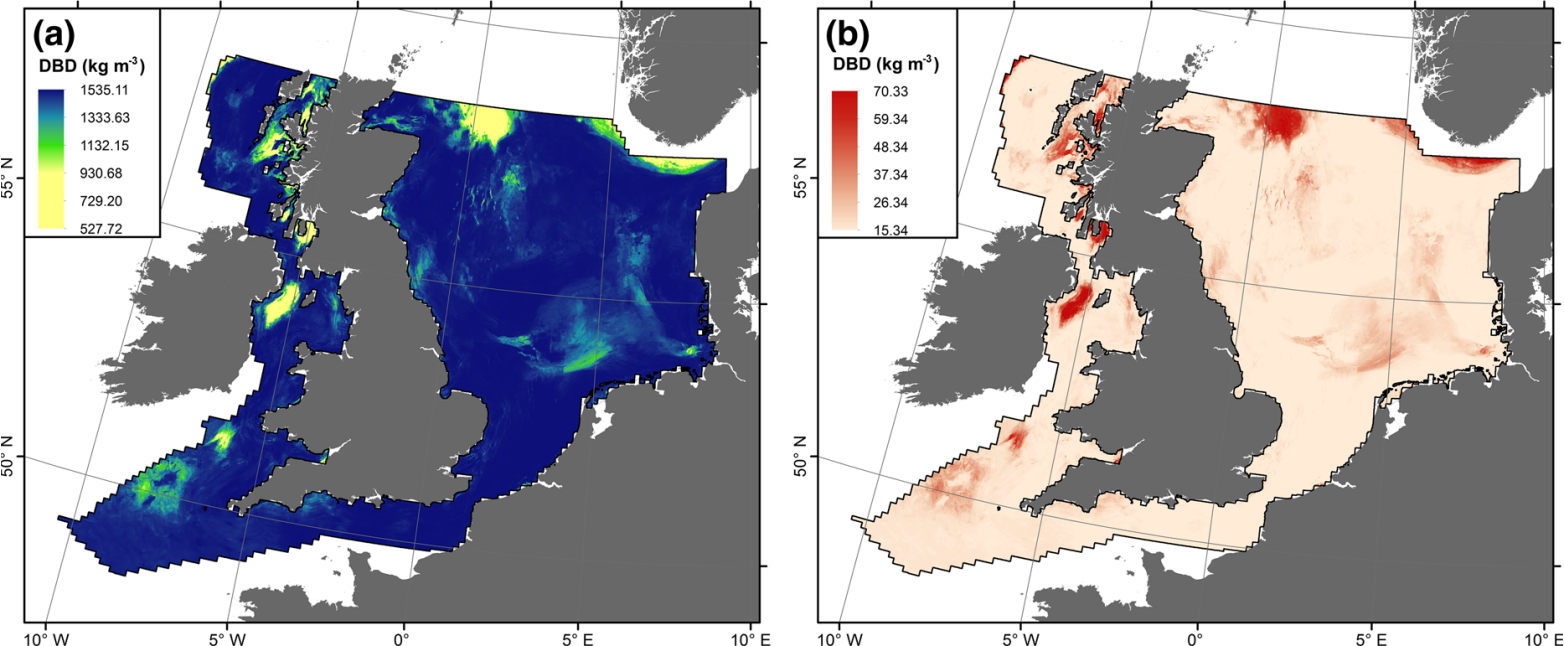
**a** Dry bulk density based on Eq. 5 for porosity estimation. **b** Difference between dry bulk density based on Eq. 5 for porosity estimation and dry bulk density based on Eq. 8 for porosity estimation

However, Eq. 5 systematically yields slightly lower estimates for sediment porosity and consequently slightly higher estimates of dry bulk density (Fig. 6). Differences are highest in muddy basins.

### POC in surficial sediments

**Fig. 7:**
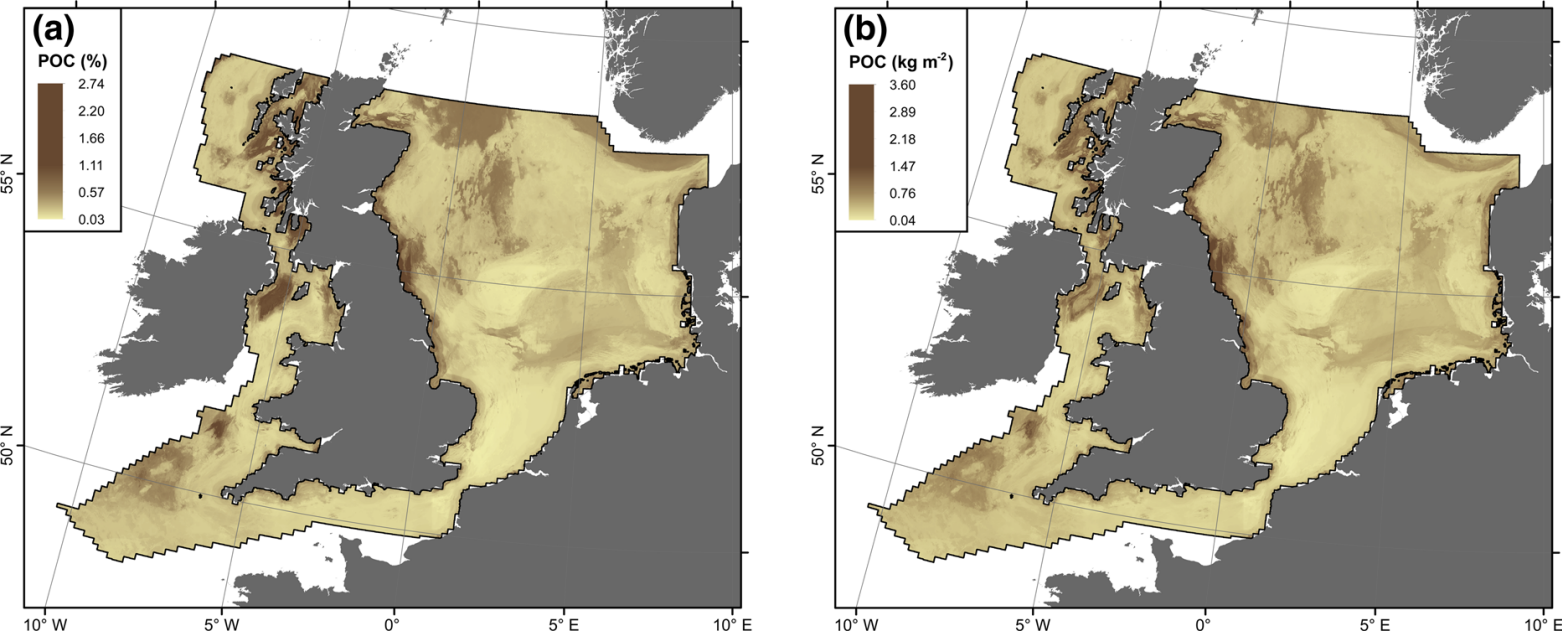
**a** Predicted concentrations of POC across the study site; **b** Predicted mass of POC per unit area seabed to a depth of 10 cm

Spatial patterns of POC are shown in Fig. 7. Highest POC concentrations are associated with the Norwegian Trench, shelf basins and coastal areas around Scotland and north–east England. Lowest concentrations are found in the southern North Sea, on Dogger Bank, the English Channel and in the deeper parts of the Irish Sea. Dry bulk density is negatively correlated with mud content. Areas of high mud content, mainly the shelf basins, consequently have a low dry bulk density and vice versa (Fig. 6). This means that the highest mass of POC per unit area (m^2^) is associated with coastal areas around Scotland and north-east England and the rims of shelf basins. Using Eq. 5 for the estimation of porosity yields a total standing stock of POC in the top 10 cm of shelf sediments of m_POC_ = 247.1 Mt over an area of 632,881 km^2^, equal to 390.4 t of POC per 1 km^2^. Alternatively, using Eq. 8 for the estimation of porosity yields m_POC_ = 243.3 Mt, which is 3.8 Mt (1.5%) lower than the previous estimate. Due to these limited differences, we will subsequently use estimates of POC derived by utilising Eq. 5.

Statistical values for POC concentrations and dry bulk density by Folk sediment class are summarised in Table 4. Note that no estimates could be made for slightly gravelly mud. Statistics reported for gravelly mud and muddy gravel are based on a very limited number of data points as these sediment types rarely occur within the study site. The highest POC concentrations are associated with gravelly mud, mud and sandy mud. Conversely, gravel and sandy gravel exhibit the lowest POC concentrations. The highest standing stock of POC is associated with sand due to the large area and high dry bulk density. Sediments with the highest POC concentrations provide a minor contribution to the overall POC stock. The total standing stock of POC estimated from mean values per sediment class as reported in Table 4 is m_POC_ = 252.2 Mt, which is slightly higher (2%) than estimated by summing up predicted values as reported above.

**Table 4:** Statistical values for POC concentrations and dry bulk density by Folk sediment class

Folk class	Area (km^2^)	POC (%)				Dry bulk density (kg m^−3^)				
		P5	P95	Mean	SD	P5	P95	Mean	SD	POC stock (Mt)
Mud	3080	0.59	1.11	0.88	0.20	536	624	580	29	1.56
Sandy mud	13,656	0.54	1.11	0.78	0.21	646	1011	828	120	8.81
Muddy sand	64,043	0.27	0.92	0.54	0.22	1111	1429	1323	99	45.49
Sand	323,200	0.10	0.50	0.24	0.12	1454	1535	1511	25	116.24
Slightly gravelly sandy mud	122	0.55	0.93	0.67	0.16	789	1030	945	73	0.08
Slightly gravelly muddy sand	5772	0.32	0.82	0.54	0.22	1192	1433	1357	80	4.20
Slightly gravelly sand	92,414	0.07	0.43	0.22	0.11	1467	1534	1512	21	31.13
Gravelly mud	2	0.70	1.69	0.91	0.51	845	1080	1011	102	0.00
Gravelly muddy sand	1638	0.30	0.77	0.49	0.23	1287	1447	1397	51	1.12
Gravelly sand	90,987	0.12	0.44	0.23	0.10	1486	1534	1515	16	32.35
Muddy gravel	1	0.62	0.62	0.62	0.01	1234	1394	1314	125	0.00
Muddy sandy gravel	802	0.16	0.45	0.29	0.10	1438	1510	1482	25	0.34
Sandy gravel	35,222	0.12	0.35	0.19	0.09	1492	1534	1521	13	10.33
Gravel	1942	0.13	0.25	0.18	0.05	1511	1535	1529	8	0.55
Sum	632,881									252.21

### Scaling up to the NW European continental shelf

A simple scaling up of m_POC_ to the area of the NW European continental shelf based on the average mass of POC per unit seabed and to a depth of 10 cm yields m_POC_ = 434 Mt. Based on mean values of POC and dry bulk density (Table 4), we derive m_POC_ = 476 Mt. The lower and upper bounds of our estimates (based on the 5th and 95th percentiles) are calculated to 230 Mt and 882 Mt, respectively. Note that the contribution of slightly gravelly mud was based on statistics for the mud class, as no statistical values could be derived.

## Discussion

We have described a quantitative spatial model of POC concentrations in surficial sediments of parts of the NW European continental shelf. The results were produced with a repeatable method and validated with independent, i.e. not used for model building, sample data. The derived statistics indicate that the model is not over-fitted to the training data and more than three quarters of the variance in the response data are explained by the model. Predicted POC concentrations are highly and significantly correlated with measured POC concentrations, although the model appears to slightly under-predict POC concentrations.

### Model appraisal and limitations

Every model has limitations as it is a simplification of reality and this is also true in this case. In the following, we will discuss the major limitations of our RF model:

Firstly, care was taken to include as many potentially relevant predictor variables as possible. Initially, a conceptual model was developed and potentially relevant variables were identified. Subsequently, those were selected for which full coverage information of the study site was available. These were then subjected to a formal variable selection process. Important variables may be not included in the final model due to two reasons: (i) important additional variables may have been omitted when creating the conceptual model or (ii) it was not possible to obtain data on a variable with full coverage. The latter is especially likely to have occurred as at the time of model development we were not able to source an even more comprehensive set of physico-chemical, chemical and biological variables including water column and porewater nutrients concentrations, oxygen saturation in bottom waters and sediment pH distributions. Furthermore, the inclusion of biological variables such as sediment microbial or faunal communities was beyond the scope of this stage of model development. However, such variables might be accounted for to a certain extent indirectly via correlated variables included in the model.

We believe that further improvements in model performance are generally possible through a better understanding of causal relationships (e.g. the processes by which temperature or distance to shoreline might affect carbon storage and mineralisation) leading to better conceptual models underpinning the applied statistical approaches, through additional data on relevant predictor variables becoming available as a consequence of advances in parameter measurement and increases in spatial coverage of relevant observations. However, we believe that under the given circumstances our model is a significant achievement as indicated by the fact that 78% of the variance in the response variable is explained by the model.

The availability of predictor and response variables is an external constraint that will influence model performance. However, even if data on a variable exist in principle, they might relate to a certain time interval when the data were collected or for which they were modelled (e.g. hydrodynamic models). Additionally, predictor variables are gridded to a certain spatial resolution. In our model, mismatches between variables do exist both temporally and spatially: e.g. samples were taken from 1996 to 2015, but peak wave orbital velocity was modelled for the period 1999–2008 (Bricheno et al. 2015) and annual average bottom temperature refers to a climatology for the years 1971–2000 (Berx and Hughes 2009). The implicit assumption of our model therefore is that the response and predictor variables are constant through time. Such an assumption is unlikely to hold. However, we note that the mentioned time intervals do at least partially overlap and it might be assumed that changes in predictor variables are likely to act on longer time scales before they become significant for our model.

With regard to the spatial resolution of the gridded predictor variables we note that four out of six (distance to shoreline, eastings, mud and gravel) existed at the same resolution as the POC model. Annual average bottom temperature was provided at a lower resolution of approximately 10 km and was interpolated to match the 500 m grid of the other predictor variables. We believe that this approach is defendable as bottom temperature is likely to exhibit relatively gradual changes over distances of 10 km. Regarding peak wave orbital velocity, we obtained modelled surface wave properties (significant wave height and zero crossing period) at a spatial resolution of approximately 12 km and calculated orbital velocities at the seabed utilising a high-resolution bathymetry (c. 200 m) in an attempt to better account for small-scale variability in the predictor variable. Again, the assumption was that surface wave properties change gradually over distances of 12 km (at least in water depths beyond the wave base) and that fine-scale variability in orbital velocity is mainly driven by changes in bathymetry, for which high-resolution data existed. We are thus confident that we have accounted for the differing spatial resolutions of the predictor variables in an adequate way.

### Factors controlling POC

Mud content, annual average water column bottom temperature, eastings, distance to shoreline, gravel content and peak wave orbital velocity were identified as important predictor variables. Our results demonstrate that mud content in surficial seabed sediments is the most important variable in predicting POC concentrations, which increase with an increase in mud content. Such general relationships have been observed before in North Sea sediments (Cadée 1984; Lohse et al. 1996; de Haas et al. 1997; Trimmer et al. 2005); however, these were often based on a limited number of samples in a spatially restricted area and such studies focused on other sediment mechanisms rather than regional scale POC-sediment composition relationships directly. Various mechanisms have been proposed, including sorption of organic matter to mineral surfaces and its subsequent concentration in fine-grained sediments with large surface areas (Keil and Hedges 1993), preservation under anoxic conditions in a static situation (e.g. Black Sea and Baltic Sea) and high primary productivity in a dynamic system (coastal upwelling primarily on the western continental margins). Studies of sediment biogeochemistry and carbon cycling have often noted this relationship between sediment type and POC concentration, both in terms of POC driving remineralisation processes and towards an improved understanding of conditions which control POC stocks. Again, these studies have been geographically constrained and focused on carbon or nutrient cycling directly so have not described the regional scale relationships across shelf sea areas.

When critically evaluating the observed link between mud and gravel content and POC concentrations, it is useful to consider the two end members of such sediments and the spectrum of sediments between. A relationship of increasing POC concentrations with mud content is expected given previous findings. Fine sediments are often associated with high natural organic matter loading due to proximity to terrestrial inputs, sedimentary hydrographic environments of low natural disturbance or create an environment where POC that is deposited naturally accumulates due to the diffusional environment that the higher mud percentage creates. This favours a more reducing environment within the sediments and anoxic bacterial processes of remineralisation, which may lead to increased carbon storage in locations where anaerobic degradation processes based on alternative electron acceptors such as nitrogen, iron, manganese or sulphur are dominant. Conversely, substrates with lower mud percentages have more open structures where advective flow in the upper layers deepens the oxic layers and so POC drawn into the sediments is more rapidly respired (Huettel et al. 1996; Huettel and Rusch 2000; Ehrenhauss et al. 2004). The transitions between these two end members which control remineralisation rates through sediment and oxygen are rarely described at a regional scale and so this study bridges the gaps in understanding of the interplay between these end-point mechanisms and its relevance to POC stocks.

Similarly, the observed trend of an increase in POC concentrations with decreases in average bottom water temperature in the range of 7–12 °C covered by the observational data and model agrees with many studies on the remineralisation of POC in marine sediments, both seasonal and geographical (Berner 1980; Middelburg 1989; Burdige 1991). The decrease may be forced by the temperature dependency on bacterial processes (both oxic and anoxic), as remineralisation occurs faster at higher temperatures or alternatively by a negative relationship between primary production (supply level and POC lability) and temperature in the study area. A noticeable step change at ca. 8 °C might potentially correlate to the onset of additional metabolic pathways or non-linear temperature controls involved in POC remineralisation or additionally conditions of these low-temperature shelf areas with disproportionately slow POC remineralisation, for example refractory terrestrial organic matter (TOM) inputs and low oxygen regimes in sea-lochs or deep stratified regions (Glud 2008; Burrows et al. 2014).

The observed influence of the distance to a shoreline on POC concentrations might indicate a change in POC sources: Close to the shore, terrestrial inputs (such as drainage of peats or river catchments) and benthic primary production (e.g. Duarte et al. 2005), where benthic systems lie within the euphotic zone, might dominate measured nearshore POC concentrations. Further offshore, water column primary and secondary productivity or detrital dominated POC sources are more likely to prevail. The input of TOM within these areas can also illustrate the significance of this pool of POC closer to shore. It is a more refractory component of POC and therefore will accumulate in the stock to a greater extent than more labile marine derived POC (Burdige 2005) Chlorophyll-a concentration, which is a proxy for pelagic primary productivity, was not found to be an important predictor. This might be explained by the overriding dominance of other physical or physico-chemical determinants, variabilities in transfer of POC production within the water column and transfer to the bed (sinking, recycling) or significant lateral transport by currents during the sinking process. The derived chlorophyll-a concentrations might also be confounded by POC sources not quantified well by satellite systems (for example deep chlorophyll maxima within stratified regions) or by coloured dissolved organic matter, which is frequently found in coastal waters but was not explicitly accounted for when deriving products from the MODIS data (e.g. Gohin et al. 2005).

### POC stocks in surface sediments of the NW European continental shelf

The estimation of the POC stock (Eq. 7) also depends on robust estimates of dry bulk density. We used Eq. 5 (Jenkins 2005) to estimate sediment porosity and subsequently dry bulk density (Eq. 6). Equation 5 is based on a large data set; however, the samples were collected on the Mississippi–Alabama–Florida shelf. We have demonstrated that this relationship generally holds for our study area and that estimates of the POC stock are hardly affected. We are thus confident that our estimates of the mass of POC stored in surficial sediments of the NW European continental shelf are realistic.

Our results (Figs. 4a, 7a) support the concept that the highest concentrations of POC are associated with muddy sediments. However, these do not always translate into the highest values in terms of mass per unit area, as dry bulk densities of muddy sediments are usually low (Fig. 6). Counterintuitively, muddy sediments (mud, slightly gravelly mud, slightly gravelly sandy mud, sandy mud, gravelly mud) contribute little to the total stock due to their spatially restricted areas and low dry bulk densities. Conversely, sand, slightly gravelly sand and gravelly sand contribute 71% of the POC stock due to high dry bulk densities and widespread occurrence in the study area (Table 4). Hence, our results challenge the view that POC is mainly stored in soft, fine-grained sediments. These results indicate that future research needs to consider previously under-studied coarse-grained sediments with low mud contents.

So far, estimates of POC in the NW European shelf area only exist for localised areas (Serpetti et al. 2012; Burrows et al. 2014). Previous shelf wide approaches to carbon budgets have been carried out but have focussed on annual sedimentation rates in order to generate annual carbon budgets (de Haas et al. 1997, 2002). This latter approach, although key to our understanding of the autotrophy versus heterotrophy of shelf seas and overall carbon cycling, does not address the fundamental question of the overall stock. The results presented here are therefore the first large scale estimate of POC in this area from field samples. Our estimate of 250 Mt carbon stored in surficial sediments of the study area (A = 633,000 km^2^) contrasts with an estimate of 18.1 Mt stored in the top 10 cm of marine sediments off Scotland covering an area of approximately 470,000 km^2^ (Burrows et al. 2014), an order of magnitude lower than our estimate. Given that there is significant spatial overlap between the two study areas, it is likely that the observed discrepancy relates to differences in the approaches taken to derive the carbon stocks. Burrows et al. (2014) derived their estimate by combining average POC concentrations for different sediment types taken from published studies with the estimates of the spatial extent of these sediment types. POC concentrations were assumed to be zero for coarse-grained sediments. This might to some extent explain the discrepancies between the two estimates.

Our figure of 476 Mt (230–882 Mt) for the NW European continental shelf shows the stock to be important for example in comparison to estimates of 9000 and 25,000 Mt of carbon for the whole of Europe’s forest vegetation and soils respectively (Kauppi et al. 1992; Dixon et al. 1994). It is important to note that the area of the NW European continental shelf is 1,111,812 km^2^ in comparison to 2,830,000 km^2^ for forest cover, i.e. ~39% of the surface area for forest. Shelf sea sediments therefore have an aerial storage of ca 3.6% that of terrestrial soils in Europe showing that this store albeit smaller is nonetheless important.

We have identified key variables influencing the distribution of POC but now that we have a baseline of POC we can in the future address variables that influence change in this stock. Long term changes in water column variables such as light penetration (Capuzzo et al. 2015) and changes in anthropogenic physical pressures such as beam trawling intensity (Callaway et al. 2007) may be important in terms of the overall primary production reaching the bed and sediment storage. Additionally, potential changes in terrestrial land-use and catchment management policies are likely to alter inputs of terrestrially derived POC. The maps (Fig. 7) we have presented may therefore be used in the future to show how changes of these and other variables alter the sediment stock. It is likely that the factors identified here in controlling regional POC stock (temperature, terrestrial inputs, sediment composition) will all act in the future via climate change or through human activities to alter these stocks. The implications for spatially restricted muddy substrates with high POC concentrations and for the spatially extensive sand/gravel substrates with low POC concentrations are likely to be contrasting and different. These changes are important since management decisions on the distribution of fishing effort (Duineveld et al. 2007) or marine aggregate extraction (Desprez 2000) might be able to increase the storage capacity of key areas such as in coastal zones where POC stocks are at their highest. The potential and importance of coastal sediments for carbon sequestration has so far rarely been investigated. Current research has focussed on coastal vegetated habitats such as salt marsh (Chmura et al. 2003), sea grass (Fourqurean et al. 2012) and macroalgae (Krause-Jensen and Duarte 2016). Our results suggest that coastal sediments and their role in carbon storage and sequestration deserve more attention in the future.

Human activities may result in net release of CO_2_ due to sediment resuspension, or conversely an increase in POC stocks by shifting sediments which normally process carbon quickly towards regimes which favour POC storage, with consequences for the overall autotrophic/heterotrophic balance of shelf seas. The link between spatial significance of stock levels and factors that interact geographically to produce this stock and the magnitude and direction of change under future conditions or pressures will be key to future management of carbon stocks. The maps of POC stored in surface sediments presented here with the variables (e.g. mud content and bottom temperature) identified in the model provide a starting point to identify controls and vulnerabilities of present day stocks.

The magnitude of the stock on the NW European continental shelf and by extension in other temperate shelf seas shows how important the management of this stock may be globally. Information on POC stocks, their spatial distribution and the factors controlling them will allow improved management of shelf sea areas into the future or prediction of changes in POC storage as a response to climate change. We now need to apply these methods more widely to estimate the stock of all temperate shelf seas so that their importance is quantified and recognised.

## Conclusions

We have presented a method that allows to spatially predict and quantify POC stored in surficial sediments of shelf seas. We show that the surface sediments of the NE European continental shelf are a regionally important POC store. Conversely to expectations, the largest POC stocks are associated with coarse-grained sediments. We highlight the previously overlooked importance of coastal sediments as a store for and site of carbon sequestration. Key variables influencing POC concentrations in shelf sediments of the NW European continental shelf are mud content, annual average bottom temperature and distance to coastline. The resulting model outputs might be useful for a variety of purposes including assessing surface sediment carbon stores by benthic habitat and location, typifying different habitats in terms of their expected surface POC concentration and mass and evaluating their corresponding vulnerability to disturbance. Such potential future uses of the presented results would not only improve our scientific understanding of likely sediment surface POC distributions but could also form valuable information for marine policy development and management decisions.

## Electronic supplementary material

Below is the link to the electronic supplementary material.

Supplementary material 1 (PDF 721 kb)
