# Supplementary material for: Predicting the standing stock of organic carbon in surface sediments of the North–West European continental shelf
Source: Biogeochemistry. 2017 Feb 15;135(1):183–200. doi: 10.1007/s10533-017-0310-4 (PMC6961524; doi:10.1007/s10533-017-0310-4)
Supplement: Supplementary file 1 — Supplementary material 1 (PDF 721 kb) [file 10533_2017_310_MOESM1_ESM.pdf]

Estimation of the spatial extent of sediment types on the North-West European continental shelf

For the purpose of upscaling the results of the spatial predictions of particulate organic carbon (POC) stored in the top 10 cm of shelf sediments in the study area it was necessary to know the spatial extent of Folk (1954, 1980) sediment types on the North-West (NW) European continental shelf. Although Stephens and Diesing (2015) recently presented a map of Folk sediment classes for a large part of the NW European continental shelf, significant parts of the NW European continental shelf are not covered by their map. These gaps cannot be closed with existing data products, e.g. EMODnet-Geology (http://www.emodnet.eu/geology) seabed substrate maps do not have the required thematic resolution. Hence, we have derived our own purpose-built Folk sediment map and describe the process and results in the following.

The long-term, collaborative project dbSEABED (see Goff et al., 2008; Jenkins, 2002) was employed to produce a map of sediment classes across the NW European Shelf area. dbSEABED is an information processing system that uses various mathematical methods to extract harmonised, quality controlled attributes from a large, structured archive of legacy-data analyses and descriptions of sediments. Analytical numerical data is simply extracted after quality assurance. The word-descriptive data is parsed and analysed as described in Jenkins (1997), again with quality checks, which have also included validations of the process. In cases where a water depth value was not available, that was attached using the SRTM 30-Plus global bathymetry (Becker et al., 2009). Note that skeletal biological components in the sediments, such as coral and shell, are counted in the gravel proportions.

A sub-set of 74,046 data points for the area (Figure S1) was projected to ETRS89-LAEA coordinates. The median separation of data points is 1.4 km and the median distance from any ocean-area cell to nearest data is 20.0 km (mostly due to deep water sample spacings). Kilometre scale cell-wise values for the average and standard deviation of gravel, sand and mud contents were calculated for the data-bearing cells, then predicted for vacant cells within a 20 km radius (less as the shore is approached). The prediction was carried out by local Inverse Distance Weighting (IDW; see Tomczak, 1998 for geographic distances) with a filter against data more than a factor of 2 different in water depths from the target cell's depth. This is important for mapping inshore as well as shelf-break and continental slope areas. Vacant parts of the map beyond the reach of this process were filled using a Random Forest (RF; Breiman, 2001) predictor, employing the variables water depth, bathymetric gradient and standard

deviation and distance to shoreline. After merging these, Folk codes (BGS variant) were computed from the gridded textural values. Uncertainties on the data were computed rigorously in the local IDW process, but were assigned to 100% of parameter range for the global RF process. Discrepancies due to ‘compositional data’ effects (Aitchison, 1986) were placed in the uncertainties.

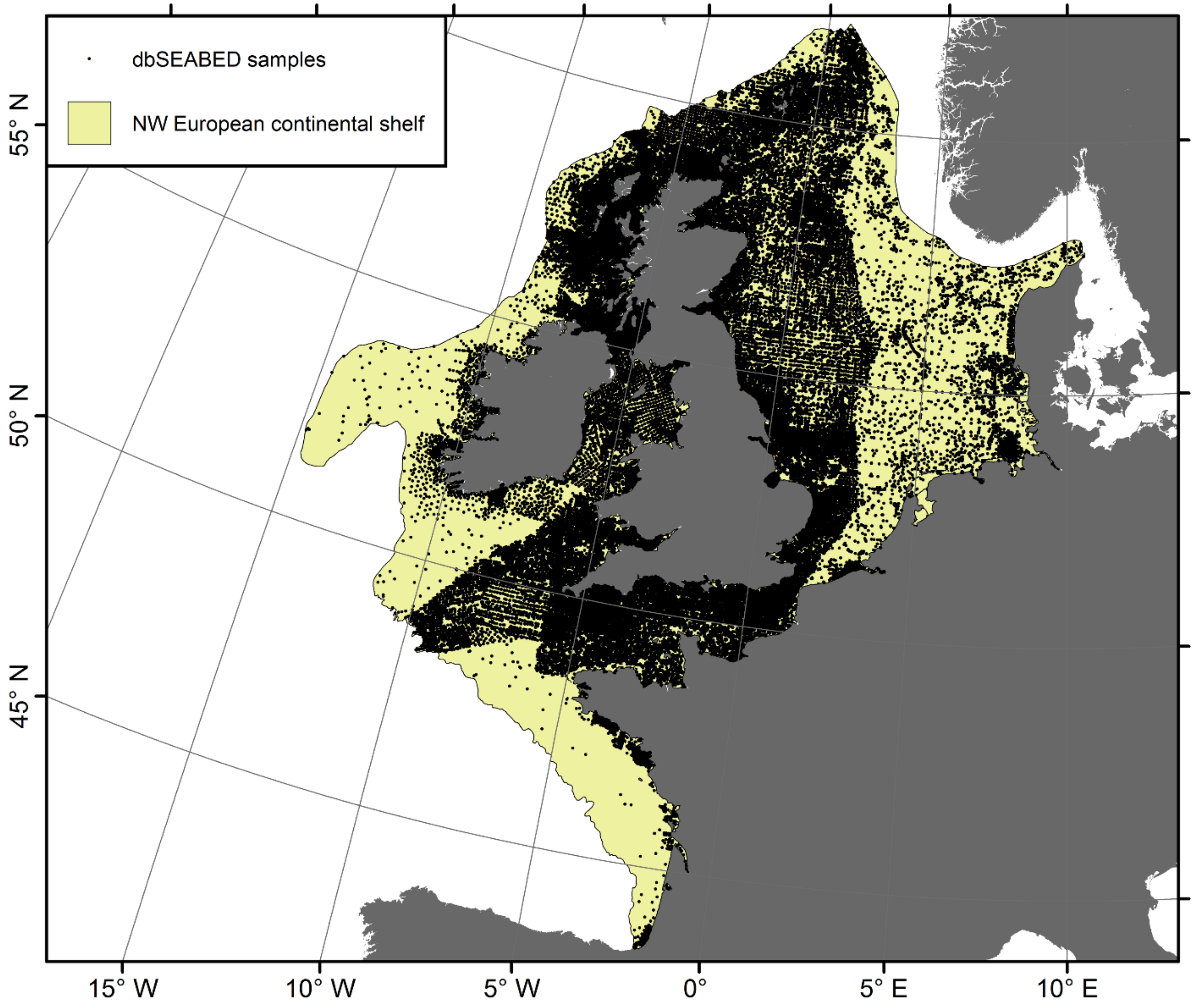

Fig. S1 Locations of samples from dbSEABED utilised to predict sediment composition

Outputs of the spatial predictions were combined with the map of Stephens and Diesing (2015) to derive a map of Folk sediment classes for the NW European continental shelf (Figure S2). The spatial extent of the different sediment classes was extracted and is detailed in Table S1.

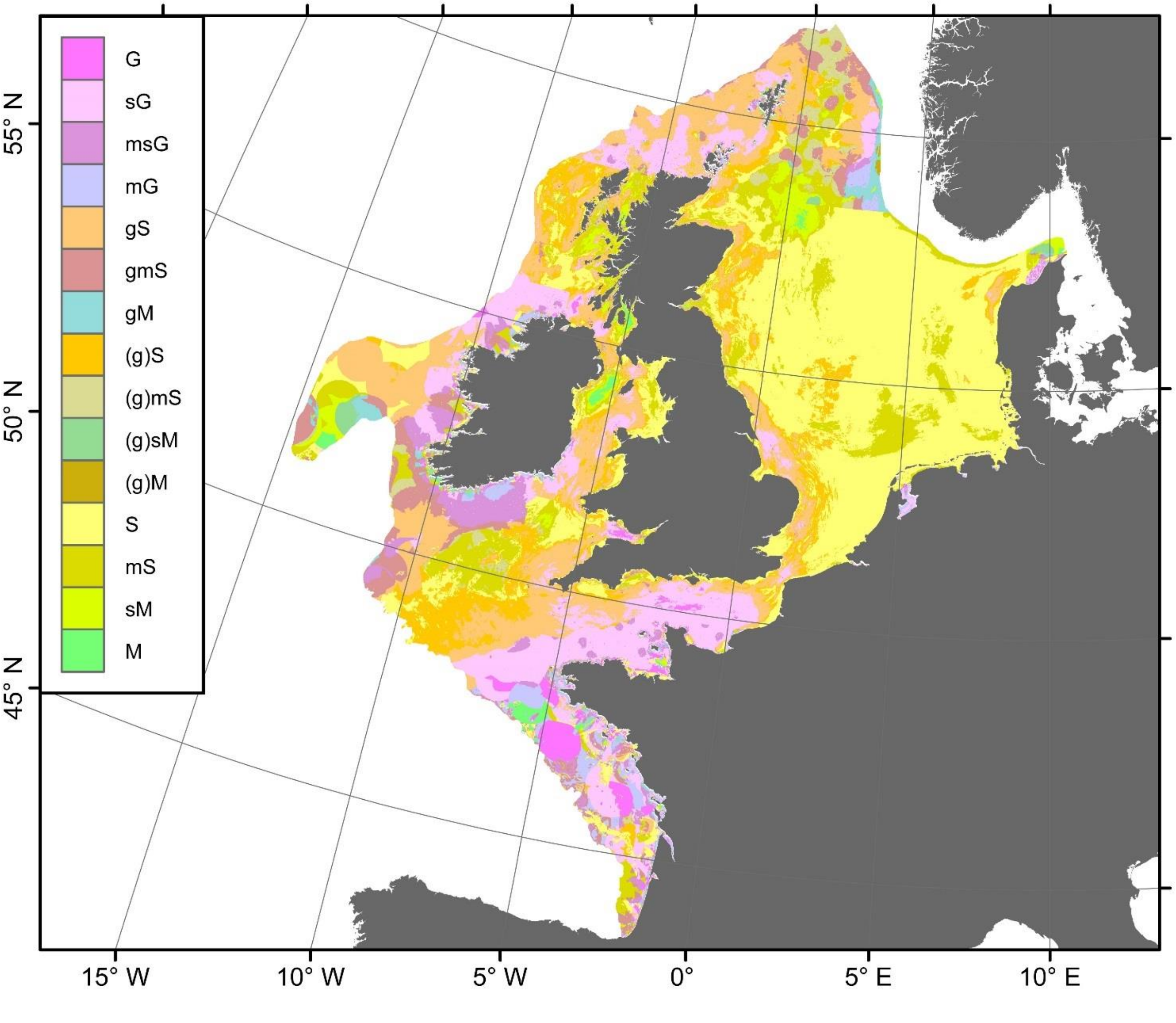

Fig. S2 Predicted map of Folk sediment classes on the NW European continental shelf. G – gravel; sG – sandy gravel; msG – muddy sandy gravel; mG – muddy gravel; gS – gravelly sand; gmS – gravelly muddy sand; gM – gravelly mud; (g)S – slightly gravelly sand; (g)mS – slightly gravelly muddy sand; (g)sM – slightly gravelly sandy mud; (g)M – slightly gravelly mud; S – sand; mS – muddy sand; sM – sandy mud; M - mud

Table S1: Estimated spatial extent of Folk sediment classes on the NW European continental shelf.

Folk class	Area (km^2)
**M**	8626
**sM**	22277
**mS**	96327
**S**	357075
**(g)M**	496
**(g)sM**	4540
**(g)mS**	28191
**(g)S**	111266
**gM**	13422
**gmS**	50217
**gS**	191909
**mG**	24184
**msG**	36914
**sG**	145122
**G**	21247
**Sum**	1111812

References

Aitchison, J.: The Statistical Analysis of Compositional Data, Book, Chapman and Hall, London., 1986.

Becker, J. J., Sandwell, D. T., Smith, W. H. F., Braud, J., Binder, B., Depner, J., Fabre, D., Factor, J., Ingalls, S., Kim, S.-H., Ladner, R., Marks, K., Nelson, S., Pharaoh, A., Trimmer, R., Von Rosenberg, J., Wallace, G. and Weatherall, P.: Global Bathymetry and Elevation Data at 30 Arc Seconds Resolution: SRTM30_PLUS, Mar. Geod., 32(4), 355–371,

doi:10.1080/01490410903297766, 2009.

Breiman, L.: Random Forests, Mach. Learn., 45(1), 5–32, 2001.

Folk, R. L.: The distinction between grain size and mineral composition in sedimentary-rock nomenclature, J. Geol., 62, 344–359, 1954.

Folk, R. L.: Petrology of sedimentary rocks, Book, Hemphill Publishing Company, Austin, Texas., 1980.

Goff, J. A., Jenkins, C. J. and Jeffress Williams, S.: Seabed mapping and characterization of sediment variability using the usSEABED data base, Cont. Shelf Res., 28(4), 614–633, doi:10.1016/j.csr.2007.11.011, 2008.

Jenkins, C.: Automated digital mapping of geological colour descriptions, Geo-Marine Lett., 22(4), 181–187, doi:10.1007/s00367-002-0111-0, 2002.

Jenkins, C. J.: Building Offshore Soils Databases, Sea Technol., 38(12), 25–28, 1997.

Stephens, D. and Diesing, M.: Towards quantitative spatial models of seabed sediment composition, PLoS One, 10(11), e0142502, 2015.

Tomczak, M.: Spatial Interpolation and its Uncertainty Using Automated Anisotropic Inverse Distance Weighting (IDW) - Cross-Validation/Jackknife Approach, J. Geogr. Inf. Decis. …, 2(2), 18–30, 1998.
